# Examining the Cervical Screening Behaviour of Women Aged 50 or above and Its Predicting Factors: A Population-Based Survey

**DOI:** 10.3390/ijerph13121195

**Published:** 2016-12-02

**Authors:** Carmen W. H. Chan, Kai Chow Choi, Rosa S. Wong, Ka Ming Chow, Winnie K. W. So, Doris Y. P. Leung, Wendy W. T. Lam, William Goggins

**Affiliations:** 1 The Nethersole School of Nursing, The Chinese University of Hong Kong, New Territories, Hong Kong, China; kchoi@cuhk.edu.hk (K.C.C.); szemanwg@hotmail.com (R.S.W.); kmchow@cuhk.edu.hk (K.M.C.); winnieso@cuhk.edu.hk (W.K.W.S.); dorisleung@cuhk.edu.hk (D.Y.P.L.); 2 School of Public Health, The University of Hong Kong, Hong Kong, China; wwtlam@hku.hk; 3 School of Public Health and Primary Care, The Chinese University of Hong Kong, New Territories, Hong Kong, China; wgoggins@cuhk.edu.hk

**Keywords:** cervical cancer, screening behaviour, protection motivation theory, Chinese women, population-based study, menopause

## Abstract

Under-screening may increase the risk of cervical cancer in middle-aged women. This study aimed to investigate cervical cancer screening behaviour and its predictors among women aged 50 years or above. A population-based sample of 959 women was recruited by telephone from domestic households in Hong Kong, using random methods, and a structured questionnaire developed to survey participants. Multivariable logistic regressions were performed to examine the factors independently associated with cervical screening behaviour. Nearly half the sample (48%) had never had a cervical smear test. Multivariable analyses showed that age, educational level, marital status, family history of cancer, smoking status, use of complementary therapy, recommendation from health professionals, and believing that regular visits to a doctor or a Chinese herbalist were good for their health were predictors of cervical screening behaviour. Misconceptions concerned with menopause may reduce women’s perceived susceptibility to cervical cancer, especially if they are 50 or above, and exert a negative effect on their screening behaviour. Healthcare professionals should actively approach these high-risk groups–older unmarried women, smokers, those less educated and who are generally not much concerned with their health.

## 1. Introduction

Cervical cancer is one of the most preventable cancers in women. Since the disease is usually diagnosed in middle age, it is important for women aged 50 or above to undergo regular screening. Population-based cervical screening programmes have been developed in North America and Europe and achieved high screening rates and significant reductions in cervical cancer deaths [1,2,3]. During the past decade, major organisations in the United States and here in Hong Kong have consistently argued against routinely screening women aged 65 or above with a history of normal screening results [4], so that they are not harmed by unnecessary procedures. However, women under 65 are still highly susceptible to the disease, and recommendations not to screen those over 65 may lead to under-screening among some middle-aged older women (between 50 and 65) who should be screened more often than they are. 

Public health researchers often utilise components of motivation theories to improve health. The Protection Motivation Theory (PMT) is an example, a cognitive theory commonly used to explain health behaviour [5,6]. Protective health behaviour occurs when one perceives risk (threat appraisal) and believes that preventive behaviour is effective (coping appraisal) [5,6]. Empirical and theoretical studies on cervical cancer screening have identified knowledge and perceptions of the personal risk of cervical cancer as important factors contributing to screening attendance [7]. Women who perceive a higher risk of developing cancer are more likely to seek preventive screening [8,9]. However, the evidence shows that Asian women’s knowledge of cervical cancer is inadequate [10,11,12]. A study conducted in mainland China found that 71% of participants underestimated the risk of cervical cancer after menopause, with higher screening rates only observed among women who understood the procedure well [10]. However, another study examining the screening behaviour of mainland Chinese women found that risk perception was not associated with screening uptake [13]. It is possible that in collectivistic cultures motivation to have a cervical smear is affected not only by perceptions of personal risk but also by advice from others, such as a spouse or healthcare professional. 

Hong Kong is a unique place, permeated by ancient Chinese culture while strongly influenced by contemporary Western values in everyday life. The perceptions and practices of health behaviour form a notable instance of this unique quality—for example, the combined use of Western and traditional Chinese medicine, and the concomitant desire for individualism and social obligation inherited from Confucianism [10]. The effect and interpretation of risk perceptions on protective health behaviour underscore the complexity of the motivational process leading to Hong Kong women’s decision to seek cervical screening. Although previous research has examined the cervical screening practices of young women in Hong Kong [14,15], there is only limited understanding of the knowledge and attitudes of older Chinese women. Given that middle age is both a risk factor of cervical cancer and a predictor of not having undergone screening, there is a potential to improve cervical cancer mortality rates through approaching women aged 50 or above who are under-screened. In order to achieve such an end, more research effort is required for a better understanding of the factors contributing to screening attendance among women in this age group. 

The present study therefore aimed to use a population-based telephone survey to assess the uptake pattern of cervical cancer screening among Chinese women aged 50 or above, and to identify predictors prompting this population to undergo cervical smear tests. 

## 2. Materials and Methods 

### 2.1. Study Design, Setting and Participants

This was a sub-sample of 1002 women from a large scale cross-sectional population-based telephone survey, using random sampling, conducted among Hong Kong Chinese women aged 50 or above. Domestic households were selected, by computer-assisted randomisation, from up-to-date residential telephone directories covering 95% of Hong Kong households. A woman respondent from each household selected who met the following criteria was invited to participate in the survey: (1) of Chinese ethnicity; (2) aged 50 or above; (3) community-dwelling; (4) able to speak Cantonese; (5) with no history of cervical cancer. In order to avoid over-representing the non-working population, the telephone interviews were conducted from 6:30 p.m. to 10:30 p.m. In the case of non-responding households, three more attempts were made to make contact, in each of the morning, afternoon and evening time slots and on another day of the week, before “non-contact status” was assigned. This practice aimed to minimise non-response bias [16]. In households with more than one eligible respondent, the member whose birthday was closest to the interview date was invited to participate. Each eligible respondent was briefed about the survey and verbal consent obtained before it began. 

### 2.2. Questionnaire

A structured questionnaire developed from the 2005 National Health Interview Survey cancer module [17] was used for the survey. It consisted of five parts: socio-demographic characteristics, health status and family history of cancer, perceived susceptibility to cancer and other health-related perceptions, utilisation of complementary medicine, and utilisation of cervical cancer screening. Part One focused on socio-demographic characteristics, including age, educational level, marital status, employment status and monthly household income. Part Two consisted of questions to identify whether the respondents had had any chronic diseases or had been diagnosed with cancer or other serious diseases in the past. Their smoking status and family history of cancer were also collected at this stage. Part Three consisted of items assessing participants’ perceptions of their health and susceptibility to cancer, as well any measures taken for the good of their health. Part Four, concerned with complementary therapies, consisted of items on five types commonly used in a Chinese society: acupuncture, cupping, herbal remedies, bone setting and Chinese massage. Each item was rated on a four-point scale (0 = never, 1 = once only, 2 = occasionally, 3 = on a regular basis), and a total index score was calculated by summing the five item scores to quantify preferences in the use of complementary therapy. The final part consisted of items examining the uptake of cervical cancer screening tests, followed by an open-ended question allowing the respondent to give her reasons for screening or not screening. “Ever having had a cervical smear test” was considered the main outcome variable of the study. Information on socio-demographic characteristics, health status and family history of cancer, perceived susceptibility to cancer and other health-related perceptions, and use of complementary medicine were treated as independent variables in this exploratory correlational study. 

### 2.3. Ethical Considerations

The study was approved by the Survey and Behavioural Ethics Committee of the Chinese University of Hong Kong (124/16). Each eligible respondent was briefed on the purposes of the survey and verbal consent was obtained before data collection. In the case of a woman declining to participate in the survey, telephone contact was terminated immediately, with no further contact being made. Respondents were told that they could terminate the telephone interview at any time and assured of the confidentiality of the data collected.

### 2.4. Statistical Analysis

Data was summarised and presented using appropriate descriptive statistics. All categorical variables were presented by their frequencies (percentages). Binary logistic regression was used to examine factors associated with the use of cervical cancer screening behaviour. The explanatory factors explored are presented in Table 1 and Table 2. A binary outcome variable was used to study screening behaviour: “Ever had a cervical smear test?” (Yes/No). Univariate analysis of the association between outcome variable and each explanatory factor was carried out by means of binary logistic regression. Those factors with *p* values < 0.25 in the univariate analyses were chosen as candidate variables for backward stepwise multivariable logistic regression to explore explanatory factors independently associated with screening behaviour outcome. The results of the significant explanatory factors identified in the multivariable logistic regression were presented by their odds ratios (OR) and 95% confidence intervals (CI). All statistical analyses were performed using SPSS 20.0 (SPSS Inc., Chicago, IL, USA). All statistical tests were two-sided and a *p*-value < 0.05 was considered statistically significant.

## 3. Results

A total of 1002 women aged 50 or above participated anonymously in the survey, and the 959 (96%) who gave complete responses to the screening behaviour items were included in the final study. 

### 3.1. Socio-Demographic Characteristics and Health Status

The socio-demographic characteristics and health status of the respondents are shown in Table 1. The mean age of the respondents was 63.3 ± 10.7 years. Fewer than half had received secondary level education or above (47%). The majority were married or cohabiting (70%), and were unemployed (85%). Thirty-two per cent of the respondents had middle (HK$10,000–29,999) to high (HK$30,000+) monthly household incomes (1US$ ≈ 7.8HK$). However, a large proportion (39%) declined to disclose, or did not know, their monthly household income. Fewer than half had a chronic illness (45%), and only 9% had ever had a serious disease or cancer. Twenty-two percent of participants had a family history of cancer, and 2% were current smokers.

### 3.2. Health Related Perceptions and Utilisation of Complementary Therapies

Table 2 shows the respondents’ health-related perceptions and utilisation of complementary therapies. The majority perceived their health status as fair or poor (62%), and most believed that exercise (77%) and maintaining a healthy diet (75%) were good for their health. About half (48%) agreed that visiting a doctor regularly was also good for health. However, only a quarter perceived that visiting Chinese herbalists regularly or taking dietary supplements was beneficial. The majority (65%) perceived that they were less susceptible to cancer and 30% of respondents were unsure of their risk. Only 12% to 35% of the respondents had ever used any of the five complementary therapies common in Chinese societies: acupuncture, cupping, herbal remedies, bone setting and Chinese massage.

### 3.3. Cervical Cancer Screening Behaviour

Among all respondents, nearly half (48%) had at some time had a cervical smear test. The three main reasons for undertaking the most recent test were: (1) regular medical check-up (77%); (2) prompted by local signs and symptoms (6%) and (3) physician’s recommendation (5%). The three most common reasons mentioned by the respondents who had never had a test were: (1) they thought a test was not necessary after the menopause (66%); (2) they thought they were healthy all along (9%), and (3) other reason not specified (5%) (Table 3). Although most of the respondents who had undergone screening did so as part of their routine health examination, only 9% had actually been recommended by a health professional for a cervical smear test. 

### 3.4. Factors Associated with Ever Having Had a Cervical Smear Test

The explanatory factors listed in Table 4 were examined for their association with ever having had a cervical smear test. Backward multivariable logistic regression using those factors with *p* values < 0.25 in univariate analysis as candidate variables revealed that screening attendance was significantly associated with all the following factors: (1) age; (2) educational level; (3) marital status; (4) family history of cancer; (5) smoking status; (6) use of complementary therapy; (7) recommendation from a health professional; (8) believing that visiting a doctor regularly was good for the health; and (9) that visiting a Chinese herbalist regularly was also good for the health (Table 4). Respondents in older age groups were less likely to have had a cervical smear test (odds ratios (OR) for women aged 60–69, 70–79 and 80 or above vs. women aged 50–59 were 0.53 (95% CI: 0.36–0.77), *p* = 0.001; 0.29 (0.18–0.44). *p* < 0.001 and 0.12 (0.06–0.23), *p* < 0.001 respectively). Women with a higher educational level were more likely to have had the test (secondary vs. primary or below, OR = 1.55 (1.10–2.19), *p* = 0.012; post-secondary or above vs. primary or below, OR = 2.59 (1.49–4.48), *p* = 0.001). Married or cohabiting respondents were more likely to be screened (OR = 1.80 (1.25–2.58), *p* = 0.001). Women who had a family history of cancer (OR = 1.89 (1.30–2.73), *p* = 0.001), and thought visiting a doctor (OR = 2.09 (1.51–2.88), *p* < 0.001) or a Chinese herbalist (OR = 1.96 (1.31–2.93), *p* = 0.001) was good for the health were all associated with increased odds of ever having had the test. Women most often using complementary therapies were more likely to have had the test than women doing so least often (OR = 2.07 (1.38–3.12), *p* < 0.001). It is of note that a recommendation from a health professional had a strong influence on screening test uptake (OR = 4.04 (2.21–7.39), *p* < 0.001). However, women with a history of smoking had decreased odds of screening attendance (OR = 0.28 (0.11–0.72), *p* = 0.008). Table 5 shows the associations between the retained variables in the multivariable logistic regression and those that showed significance in univariate but not in multivariable analysis. We found that the youngest group of women (aged 50–59), those without a family history of cancer, those who least frequently used complementary therapies and those who did not think visiting a Chinese herbalist was good for the health were less likely to perceive themselves as susceptible to cancer. 

Further subgroup analyses were conducted to identify factors associated with ever having had a cervical smear test among women aged 50–69 (Table 6). The results showed that older women aged 60–69 and those with a history of smoking were less likely to have had a test. On the other hand, women were more likely to have had a test if they had reached matriculation or a higher education level, were married, had a family history of cancer, used complementary therapy, had been recommended by a health professional for a test, or believed visiting a doctor or Chinese herbalist regularly as good for the health.

## 4. Discussion

Previous studies of Chinese and Western populations have observed that screening-related beliefs and knowledge influence women’s uptake of cervical cancer screening services [8,9,18]. In the present study, fewer than half of Chinese older women had ever had a test, a lower rate than those reported by the comparable age group in other populations [16,17,19,20,21]. The majority of these women (65%) perceived their own low susceptibility to cancer, and 30% were unsure about their risk. In line with a previous study in China [13], the present study found no association between perceived susceptibility to cancer and screening behaviour. This finding may be partly explained by Chinese older women’s lack of awareness of their risk of cancer, and of the benefits associated with early cancer screening, including that for cervical cancer [7,11,14,22]. 

On the other hand, with coping strategies and health beliefs that motivate a person to adopt healthy behaviour, married women had a higher uptake rate, consistent with previous studies among both Chinese and Western women [10,23]. This finding reveals that screening is not only a means of self-protection but is somehow prompted by social and family obligations to stay healthy. Women with positive perceptions of health-seeking behaviour (i.e., not smoking, visiting a doctor or Chinese herbalist and using complementary therapies) were more likely to attend screening. In addition, the majority of women underwent cervical screening as part of a medical checkup, indicating that organised screening programmes were useful in motivating women to attend, perhaps because they feel socially obliged to participate in the screening service. Moreover, referral by health professionals, with their medical expertise and authority, may further enhance women’s compliance and trust in the screening service [24,25]. 

**Figure 1:**
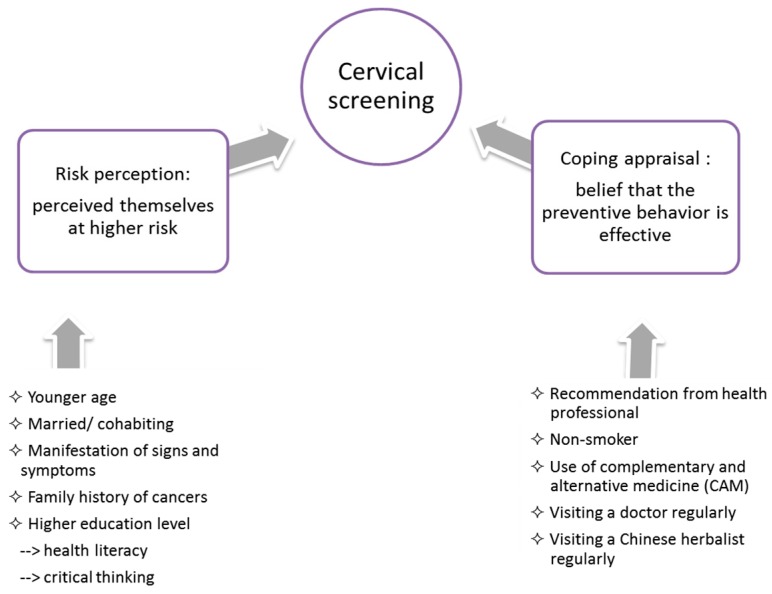
Factors associated with cervical cancer screening.

Factors affecting decisions can be categorised into risk and coping appraisal factors. The risk appraisal factors include age, sexual status, family history of cancer, manifestation of symptoms and educational level. The coping appraisal factors include health beliefs and practices such as adopting complementary therapies, recommendation from health professionals, visiting a doctor or Chinese herbalist and a history of smoking. Figure 1 is a proposed model setting out the predictive factors in cervical cancer screening. Specifically, the likelihood of having a screening test decreases with age. Over half of our respondents cited their belief that screening was not necessary after the menopause (66%) as the reason for not having a test, suggesting that Chinese women may have misconceptions about the risk of cervical cancer at more advanced ages, undermining the motivation to undergo cervical screening in women aged 50–65, even though they are still at risk of the disease. These misconceptions were also found among Western single women with a low uptake rate of cervical screening [18]. 

Our finding of an association between marital/cohabiting status and participation in screening is consistent with those of other Chinese and Western studies [10,15,23]. However, it should be noted that we did not ask our participants about their sexual activity. Respondents who were not married but still sexually active might have underestimated their risk of cervical cancer and reported low screening attendance in our study. It is important that older women or those who are single remain informed of their risk of cervical cancer, and are encouraged to undergo routine cervical screening. Healthcare professionals need to act as information providers and to understand the specific cultural factor concerned with sexual matters when delivering health information because women, especially those from Asian backgrounds, may be reluctant to discuss sexual matters openly for reasons of modesty [11,26,27,28].

Health consciousness has been suggested as a key component of screening behaviour [13,29,30]. In this study there is a higher screening rate among respondents who have a family history of cancer and those of a higher education level. These findings also suggest that better educated women tend to have a more thorough understanding and heightened awareness of their own personal risk of cancer and other diseases. Higher education levels may promote women’s cervical screening uptake by improving their health literacy [31,32] and critical thinking in understanding and correctly interpreting disease-related knowledge, including signs and symptoms [33]. This may be particularly important for Chinese populations because they do not usually go for screening until after the appearance of symptoms. Raising women’s health consciousness through communication and information provided by nurses and physicians may encourage these women to initiate preventive health checks themselves before any onset of disease. 

### Limitations

In this study, women reported their screening experiences retrospectively, which may have led to recall bias. Moreover, it was a cross-sectional study, and so long-term screening patterns and their association with knowledge and risk perception could not be explored. There were no questionnaire items on sexual activity, which meant the study was unable to reach any conclusion about the influence of sexual behaviour on screening uptake. Further in-depth investigation is clearly needed into the beliefs of post-menopausal women about cervical cancer screening, using a mixed method approach with a longer follow–up period, to confirm the essential motivational determinants of screening uptake.

## 5. Conclusions

Demographic characteristics, misconceptions about cervical cancer and health-seeking behaviour are important factors in undermining motivation for regular check-ups. The low screening rate among women aged 50 or above suggests a need to enhance health education on cervical cancer in this population, especially among those aged 50–65 who are still at risk and who are thus recommended to have routine cervical cancer screening. Misconceptions concerned with older age and menopause may reduce women’s perceived susceptibility to cervical cancer and exert a negative effect on their screening behaviour. Healthcare professionals such as nurses and other primary care providers need to make active approaches to these high-risk groups—older unmarried women, smokers, the less educated and those with generally low health consciousness.

## Figures and Tables

**Table 1:** Socio-demographic characteristics and health status of the respondents (*n* = 959).

**Socio-Demographic Characteristics**		***n* (%)**
Age (Years)	50–59	418 (43.6%)
	60–69	232 (24.2%)
	70–79	202 (21.1%)
	80 or Above	107 (11.2%)
Education Level	Primary or Below	510 (53.3%)
	Secondary	352 (36.8%)
	Matriculation or Above	95 (9.9%)
Marital Status	Single/Divorced/Widowed	284 (29.9%)
	Married/Cohabiting	665 (70.1%)
Employment Status	Unemployed	815 (85.1%)
	Employed	143 (14.9%)
Monthly Household Income (HK$)	<10,000	278 (29.4%)
	10,000–29,999	200 (21.1%)
	≥30,000	102 (10.8%)
	Don’t Know/Declined to Disclose	366 (38.7%)
**Health Status**		***n* (%)**
Chronic Illness	Any Confirmed Chronic Illness	433 (45.2%)
Serious Disease	Ever Had a Serious Disease or Cancer	81 (8.4%)
Family History of Cancer	No/Don’t Know	746 (77.8%)
	Yes	213 (22.2%)
Smoking Status	Never Smoked	925 (96.5%)
	Ex-Smoker	14 (1.5%)
	Current Smoker	20 (2.1%)

Data are presented as frequencies (%).

**Table 2:** Health-related perceptions and utilisation of complementary therapies.

**Health-Related Perceptions**		***n* (%)**
Perceived Health Status	Excellent/very Good/Good	365 (38.1%)
	Fair/Poor	594 (61.9%)
Believing that the Following Practices are Good for Health	Exercise	734 (76.5%)
	A Healthy Diet	715 (74.6%)
	Visiting a Doctor Regularly	462 (48.2%)
	Visiting a Chinese Herbalist Regularly	242 (25.2%)
	Taking Dietary Supplements	241 (25.1%)
Perceived Susceptibility to Cancer (Range: 1 = Not at All Likely to 10 = Extremely Likely)	≤5	621 (64.8%)
	>5	55 (5.7%)
	Unsure	283 (29.5%)
**Utilisation of Complementary Therapy**		***n* (%)**
Ever Used the Following Complementary Therapies	Acupuncture	174 (18.1%)
	Cupping	115 (12.0%)
	herbal Remedies	337 (35.1%)
	Bone Setting	225 (23.5%)
	Chinese Massage	153 (16.0%)
Use of Complementary Therapy Index	0 (≤50th Percentile)	446 (46.5%)
	1–2 (>50th–75th Percentile)	251 (26.2%)
	≥3 (>75th Percentile)	262 (27.3%)

Data are presented as frequencies (%).

**Table 3:** Cervical cancer screening behaviour—cervical smear test.

**Screening Behaviour**		***n* (%)**
Any Health Professional Recommended a Cervical Smear Test	No/Unsure	876 (91.3%)
	Yes	83 (8.7%)
Ever Had a Cervical Smear Test	No	500 (52.1%)
	Yes	457 (47.7%)
	Unsure	2 (0.2%)
**Among Those Who Had Ever Had a Cervical Smear Test (*n* = 457)**		***n* (%)**
Time since the Most Recent Test	<1 Year	75 (16.4%)
	1–2 Years	163 (35.7%)
	3–4 Years	99 (21.7%)
	5–6 Years	44 (9.6%)
	>6 Years	45 (9.8%)
	Can’t Remember	31 (6.8%)
Ever Had an Abnormal Test Result	No	426 (93.2%)
	Yes	29 (6.3%)
	Unsure	2 (0.4%)
The Three Main Reasons for the Most Recent Cervical Smear Test	1. Regular Medical Check-Up	351 (76.8%)
	2. Prompted by Local Signs and Symptoms *	28 (6.1%)
	3. Physician’s Recommendation	21 (4.6%)
**Among Those Who Had Not Had a Cervical Smear Test (*n* = 500)**		***n* (%)**
The Three Most Important Reasons For Not Having A Cervical Screening	1. Not Necessary	331 (66.2%)
	2. Healthy All Along	43 (8.6%)
	3. Other Unspecified Reason	24 (4.8%)

Data are presented as frequencies (%). * Having pain, bleeding, lumps or polyps.

**Table 4:** Factors associated with ever having had a cervical smear test.

Factors		Ever Had a Cervical Smear Test		OR_U_	*p*-Value	OR_A_ (95% CI)	*p*-Value
		No (*n* = 500)	Yes (*n* = 457)				
**Demographic Characteristics**		-	-	-	-	-	-
Age (Years)	50–59 (Reference)	141 (33.8%)	276 (66.2%)	1	-	1	-
	60–69	120 (51.7%)	112 (48.3%)	0.48	<0.001	0.53 (0.36–0.77)	0.001
	70–79	145 (71.8%)	57 (28.2%)	0.20	<0.001	0.29 (0.18–0.44)	<0.001
	80 or Above	94 (88.7%)	12 (11.3%)	0.07	<0.001	0.12 (0.06–0.23)	<0.001
Education Level	Primary or Below (Reference)	330 (64.8%)	179 (35.2%)	1	-	1	-
	Secondary	140 (39.9%)	211 (60.1%)	2.78	<0.001	1.55 (1.10–2.19)	0.012
	Matriculation or Above	29 (30.5%)	66 (69.5%)	4.20	<0.001	2.59 (1.49–4.48)	0.001
Employment Status	Unemployed (Reference)	450 (55.3%)	364 (44.7%)	1	-	NS	-
	Employed	50 (35.2%)	92 (64.8%)	2.27	<0.001	-	-
Monthly Household Income (HK$)	<10,000 (Reference)	180 (64.7%)	98 (35.3%)	1	-	NS	-
	10,000–29,999	81 (40.5%)	119 (59.5%)	2.70	<0.001	-	-
	≥30,000	26 (25.5%)	76 (74.5%)	5.37	<0.001	-	-
	Don’t Know/Decline to Disclose	206 (56.6%)	158 (43.4%)	1.41	0.037	-	-
Marital Status	Single/Divorced/Widowed (Reference)	194 (68.3%)	90 (31.7%)	1	-	1	-
	Married/Cohabiting	302 (45.6%)	361 (54.4%)	2.58	<0.001	1.80 (1.25–2.58)	0.001
**Health Status**		-	-	-	-	-	-
Any Confirmed Chronic Illness	No (Reference)	252 (48.1%)	272 (51.9%)	1	-	NS	-
	Yes	248 (57.3%)	185 (42.7%)	0.69	0.005	-	-
Ever Had A Serious Disease or Cancer	No (Reference)	462 (52.7%)	414 (47.3%)	1	-	NE	-
	Yes	38 (46.9%)	43 (53.1%)	1.26	0.316		-
Family History of Cancer	No/Don’t Know (Reference)	428 (57.5%)	316 (42.5%)	1	-	1	-
	Yes	72 (33.8%)	141 (66.2%)	2.65	<0.001	1.89 (1.30–2.73)	0.001
Smoking Status	Never Smoked (Reference)	473 (51.2%)	450 (48.8%)	1	-	1	-
	Ex-Smoker/Current Smoker	27 (79.4%)	7 (20.6%)	0.27	0.002	0.28 (0.11–0.72)	0.008
**Use of Complementary Therapy**		-	-	-	-	-	-
Use of Complementary Therapy Index	0 (≤50th Percentile)	285 (64.2%)	159 (35.8%)	1	-	1	-
	1–2 (>50th–75th Percentile)	139 (55.4%)	112 (44.6%)	1.44	0.022	1.35 (0.93–1.98)	0.118
	≥3 (>75th Percentile)	76 (29.0%)	186 (71.0%)	4.39	<0.001	2.07 (1.38–3.12)	<0.001
**Recommendation from Health Professional**		-	-	-	-	-	-
Health Professional Recommended the Test	No/Unsure (Reference)	481 (55.0%)	393 (45.0%)	1	-	1	-
	Yes	19 (22.9%)	64 (77.1%)	4.12	<0.001	4.04 (2.21–7.39)	<0.001
**Health-Related Perceptions**		-	-	-	-	-	-
Perceived Health Status	Excellent/very good/good (Reference)	180 (49.6%)	183 (50.4%)	1	-	NS	-
	Fair/Poor	320 (53.9%)	274 (46.1%)	0.84	0.198	-	-
Thought Exercise was Good for Health	No (Reference)	118 (52.7%)	106 (47.3%)	1	-	NE	-
	Yes	382 (52.1%)	351 (47.9%)	1.02	0.882	-	-
Believed a Healthy Diet was Good for the Health	No (Reference)	150 (61.5%)	94 (38.5%)	1	-	NS	-
	Yes	350 (49.1%)	363 (50.9%)	1.66	0.001	-	-
Thought Visiting A Doctor Regularly Was Good For The Health	No (Reference)	299 (60.3%)	197 (39.7%)	1	-	1	-
	Yes	201 (43.6%)	260 (56.4%)	1.96	<0.001	2.09 (1.51–2.88)	<0.001
Thought Visiting a Chinese Herbalist Regularly was Good for the Health	No (Reference)	422 (59.0%)	293 (41.0%)	1	-	1	-
	Yes	78 (32.2%)	164 (67.8%)	3.03	<0.001	1.96 (1.31–2.93)	0.001
Thought Taking Dietary Supplements was Good for the Health	No (Reference)	399 (55.6%)	318 (44.4%)	1	-	NS	-
	Yes	101 (42.1%)	139 (57.9%)	1.73	<0.001	-	-
Perceived Susceptibility to Cancer (Range: 1 = Not at All Likely to 10 = Extremely Likely)	≤5 (Reference)	311 (50.2%)	309 (49.8%)	1	-	NS	-
	>5	24 (43.6%)	31 (56.4%)	1.30	0.355	-	-
	Unsure	165 (58.5%)	117 (41.5%)	0.71	0.020	-	-

Reference: Reference group of the categorical variable; OR_U_: univariate odds ratio; OR_A_: odds ratio adjusted for other significant factors obtained from backward stepwise logistic regression analysis, using variables with *p*-value < 0.25 in univariate analysis as candidate variables; NS: not statistically significant in multivariate analysis; NE: not entered into multivariable analysis.

**Table 5:** Associations between the retained variables in the multivariable logistic regression and those that showed significance in univariate but not in multivariable analysis.

Variables	Employment Status ^a^	Monthly Household Income ^b^	Any Confirmed Chronic Illness ^a^	Perceive a Healthy Diet Is Good for Health ^a^	Perceive Taking Dietary Supplements Is Good for Health ^a^	Perceived Susceptibility to Cancer ^b^
Age	−0.364 (*p* < 0.001)	0.270 (*p* < 0.001)	0.249 (*p* < 0.001)	−0.038 (*p* = 0.235)	−0.056 (*p* = 0.086)	0.093 (*p* = 0.011)
Education Level	0.216 (*p* < 0.001)	0.294 (*p* < 0.001)	−0.117 (*p* < 0.001)	0.092 (*p* = 0.005)	0.094 (*p* = 0.004)	0.081 (*p* = 0.014)
Marital Status	−0.005 (*p* = 0.887)	0.222 (*p* < 0.001)	−0.175 (*p* < 0.001)	−0.016 (*p* = 0.628)	0.011 (*p* = 0.734)	0.067 (*p* = 0.120)
Family History of Cancer	0.102 (*p* = 0.002)	0.106 (*p* = 0.104)	−0.047 (*p* = 0.144)	0.042 (*p* = 0.193)	0.085 (*p* = 0.009)	0.177 (*p* < 0.001)
Smoking Status	0.031 (*p* = 0.339)	0.067 (*p* = 0.233)	0.018 (*p* = 0.571)	−0.095 (*p* = 0.003)	−0.007 (*p* = 0.832)	0.052 (*p* = 0.280)
Use of complementary Therapy Index	0.121 (*p* < 0.001)	0.148 (*p* < 0.001)	−0.018 (*p* = 0.583)	0.132 (*p* < 0.001)	0.149 (*p* < 0.001)	0.108 (*p* < 0.001)
Health Professional’s Recommendationt	0.080 (*p* = 0.013)	0.116 (*p* = 0.005)	0.026 (*p* = 0.427)	0.044 (*p* = 0.174)	0.062 (*p* = 0.057)	0.074 (*p* = 0.072)
Perceives Visiting a Doctor Regularly as Good for Health	0.003 (*p* = 0.924)	0.018 (*p* = 0.962)	0.216 (*p* < 0.001)	0.204 (*p* < 0.001)	0.142 (*p* < 0.001)	0.033 (*p* = 0.595)
Perceived Visiting a Chinese Herbalist Regularly as Good for Health	0.109 (*p* = 0.001)	0.102 (*p* = 0.019)	0.017 (*p* = 0.601)	0.114 (*p* < 0.001)	0.129 (*p* < 0.001)	0.083 (*p* = 0.037)

^a^ Spearmen correlation coefficient was calculated for both ordinal variables; ^b^ Cramer’s V calculated for either of the variables was nominal.

**Table 6:** Factors associated with women aged 50–69 ever having had a cervical smear test.

Factors		Ever Had a Cervical Smear Test		OR_U_	*p*-Value	OR_A_ (95% CI)	*p*-Value
		No (*n* = 261)	Yes (*n* = 388)				
**Demographic Characteristics**		-	-	-	-	-	-
Age (Years)	50–59 (Reference)	141 (33.8%)	276 (66.2%)	1	-	1	-
	60–69	120 (51.7%)	112 (48.3%)	0.48	<0.001	0.53 (0.36–0.78)	0.001
Education Level	Primary or Below (Reference)	129 (49.4%)	132 (50.6%)	1	-	1	-
	Secondary	113 (36.5%)	197 (63.5%)	1.70	0.002	1.45 (0.98–2.13)	0.062
	Matriculation or above	18 (23.7%)	58 (76.3%)	3.15	<0.001	2.67 (1.38–5.16)	0.004
Employment Status	Unemployed (Reference)	212 (41.8%)	295 (58.2%)	1	-	NS	-
	Employed	49 (34.8%)	92 (65.2%)	1.35	0.131	-	-
Monthly Household Income (HK$)	<10,000 (Reference)	84 (54.5%)	70 (45.5%)	1	-	NS	-
	10,000–29,999	63 (36.0%)	112 (64.0%)	2.13	0.001	-	-
	≥30,000	23 (23.2%)	76 (76.8%)	3.97	<0.001	-	-
	Don’t Know/Decline to Disclose	85 (40.7%)	124 (59.3%)	1.75	0.009	-	-
Marital Status	Single/Divorced/Widowed (Reference)	71 (53.4%)	62 (46.6%)	1	-	1	-
	Married/Cohabiting	188 (37.0%)	320 (63.0%)	1.95	0.001	2.31 (1.46–3.64)	<0.001
**Health Status**		-	-	-	-	-	-
Any Confirmed Chronic Illness	No (Reference)	152 (37.8%)	250 (62.2%)	1	-	NS	-
	Yes	109 (44.1%)	138 (55.9%)	0.77	0.111	-	-
Ever Had a Serious Disease or Cancer	No (Reference)	236 (40.1%)	353 (59.9%)	1	-	NE	-
	Yes	25 (41.7%)	35 (58.3%)	0.94	0.810	-	-
Family History of Cancer	No/Don’t Know (Reference)	214 (45.0%)	262 (55.0%)	1	-	1	-
	Yes	47 (27.2%)	126 (72.8%)	2.19	<0.001	1.81 (1.18–2.79)	0.006
Smoking Status	Never Smoked (Reference)	247 (39.3%)	382 (60.7%)	1	-	1	-
	Ex-Smoker/Current Smoker	14 (70.0%)	6 (30.0%)	0.28	0.009	0.28 (0.10–0.83)	0.021
**Use of Complementary Therapy**		-	-	-	-	-	-
Use of Complementary Therapy Index	0 (≤50th Percentile)	147 (54.4%)	123 (45.6%)	1	-	1	-
	1–2 (>50th–75th Percentile)	61 (38.9%)	96 (61.1%)	1.88	0.002	1.68 (1.06–2.66)	0.027
	≥3 (>75th Percentile)	53 (23.9%)	169 (76.1%)	3.81	<0.0001	2.51 (1.56–4.04)	<0.001
**Recommendation from Health Professional**		-	-	-	-	-	-
Health Professional Recommended the Test	No/Unsure (Reference)	249 (42.7%)	334 (57.3%)	1	-	1	-
	Yes	12 (18.2%)	54 (81.8%)	3.36	<0.001	3.38 (1.68–6.79)	0.001
**Health-Related Perceptions**		-	-	-	-	-	-
Perceived Health Status	Excellent/very Good/Good (Reference)	95 (38.3%)	153 (61.7%)	1	-	NE	-
	Fair/Poor	166 (41.4%)	235 (58.6%)	0.88	0.435	-	-
Perceived Exercise as Good for the Health	No (Reference)	74 (42.5%)	100 (57.5%)	1	-	NE	-
	Yes	187 (39.4%)	288 (60.6%)	1.14	0.467	-	-
Perceived a Healthy Diet as Good for the Health	No (Reference)	76 (49.4%)	78 (50.6%)	1	-	NS	-
	Yes	185 (37.4%)	310 (62.6%)	1.63	0.008	-	-
Perceive Visiting a Doctor Regularly as Good for the Health	No (Reference)	183 (51.4%)	173 (48.6%)	1	-	1	-
	Yes	78 (26.6%)	215 (73.4%)	2.92	<0.001	2.65 (1.81–3.87)	<0.001
Perceived Visiting a Chinese Herbalist Regularly as Good for the Health	No (Reference)	220 (47.8%)	240 (52.2%)	1	-	1	-
	Yes	41 (21.7%)	148 (78.3%)	3.31	<0.001	1.90 (1.18–3.06)	0.009
Perceived Taking Dietary Supplements as Good for the Health	No (Reference)	211 (44.1%)	267 (55.9%)	1	-	NS	-
	Yes	50 (29.2%)	121 (70.8%)	1.91	0.001	-	-
Perceived Susceptibility to Cancer (Range: 1 = not at All Likely to 10 = Extremely Likely)	≤5 (Reference)	160 (37.6%)	265 (62.4%)	1	-	NS	-
	>5	19 (41.3%)	27 (58.7%)	0.86	0.628	-	-
	Unsure	82 (46.1%)	96 (53.9%)	0.71	0.055	-	-

Reference: Reference group of categorical variables; OR_U_: univariate odds ratio; OR_A_: odds ratio adjusted for other significant factors obtained from backward stepwise logistic regression analysis using variables with *p*-value < 0.25 in univariate analysis as candidate variables; NS: not statistically significant in multivariate analysis; NE: not entered into multivariable analysis.
